# Invasive patterns of *Biomphalaria straminea* revealed by genetic mapping in the Greater Bay Area, China

**DOI:** 10.1186/s40249-025-01411-8

**Published:** 2026-01-15

**Authors:** Yue Hu, Hui Huang, Min-Yu Zhou, Yun-Fei Zhou, Hai-Mo Shen, Jun-Hu Chen, Zhi-Yue Lv

**Affiliations:** 1 Key Laboratory of Tropical Disease Control (Sun Yat-sen University), Ministry of Education, Guangzhou, Guangdong China; 2 Provincial Engineering Technology Research Center for Biological Vector Control, Guangzhou, Guangdong China; 3 NHC Key Laboratory of Tropical Disease Control, Hainan Medical University, Haikou, Hainan China; 4 National Key Laboratory of Intelligent Tracking and Forecasting for Infectious Diseases, National Institute of Parasitic Diseases, Chinese Center for Disease Control and Prevention (Chinese Center for Tropical Diseases Research), National Health Commission of the People’s Republic of China (NHC) Key Laboratory of Parasite and Vector Biology, World Health Organization (WHO) Collaborating Center for Tropical Diseases, National Center for International Research on Tropical Diseases, Shanghai, China; 5 Hainan Tropical Diseases Research Center (Hainan Sub-Center, Chinese Center for Tropical Diseases Research), Haikou, Hainan China; 6 Hainan General Hospital, Hainan Affiliated Hospital of Hainan Medical University, Haikou, Hainan China

**Keywords:** *Biomphalaria straminea*, Single nucleotide polymorphism, Population genetics, Invasion risk, Double digest restriction associated DNA sequencing, IPLEX assay, MaxEnt model

## Abstract

**Background:**

*Biomphalaria straminea*, an intermediate host of *Schistosoma mansoni*, is originally native to Brazil but has invaded southern China since 1974. Nowadays, increasing human mobility raises the risk of *S. mansoni* dissemination. Therefore, this study aims to elucidate the genetic variation and structure of *B. straminea* in China and develop molecular tools for tracing its geographic origins, which could aid in schistosomiasis prevention and control.

**Methods:**

We collected 290 *B. straminea* individuals from Shenzhen City (GDSZ, *n* = 171), Dongguan City (GDDG, *n* = 65), and Hong Kong (HK, *n* = 54). Double digest restriction associated DNA (ddRAD) sequencing was applied to genotype the samples. A subset of single nucleotide polymorphisms (SNPs) was validated by the Sequenom MassARRAY iPLEX assay. The MaxEnt model was employed to predict suitable habitats for *B. straminea* in China under current and future climate conditions.

**Results:**

Analysis of ddRAD sequencing data led to the identification of 80 high-confidence SNPs. *B. straminea* from GDSZ exhibited higher genetic diversity than those from other locations. The total observed heterozygosity (*Ho* = 0.35) was higher than the total expected heterozygosity (*He* = 0.26), resulting in a negative inbreeding coefficient (*Fis* = − 0.35), indicating that outbreeding has dominated the recent genetic history of *B. straminea*. Pairwise genetic distance (*Fst* < 0.05) and number of effective migrants (*Nm* > 4) indicated low genetic differentiation. The populations in GDSZ, GDDG and HK were genetically similar, with the first two being more closely related. Three high-quality SNPs displayed distinct geographical population specificity and could serve as geographically specific SNP markers. The MaxEnt model predicted an expansion of suitable habitats for *B. straminea* in China under future climate conditions. High invasion risk in Hainan Province, Guangxi Zhuang Autonomous Region, and Taiwan Province warrants attention.

**Conclusions:**

This study provides the first genome-wide insights into the population structure and genetic diversity of *B. straminea* in China. The populations are genetically similar, suggesting a common invasion source. Applying the geographically specific SNPs could enable rapid prediction of the geographic origin of *B. straminea* in future invasion events. Future climate conditions are likely to facilitate the spread of *B. straminea*, increasing the risk of schistosomiasis transmission in China.

**Graphical abstract:**

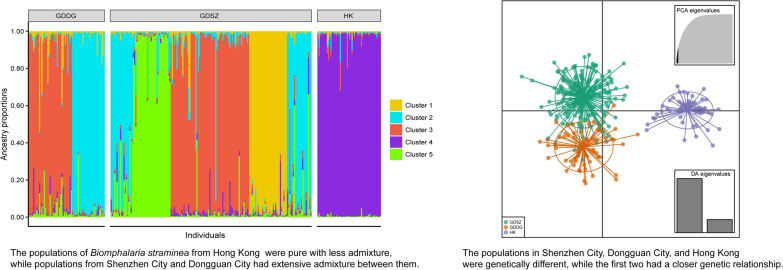

**Supplementary Information:**

The online version contains supplementary material available at 10.1186/s40249-025-01411-8.

## Background

Schistosomiasis, caused by trematodes of the genus *Schistosoma*, primarily *Schistosoma japonicum*, *S. mansoni* and *S. haematobium*, is one of the most widespread zoonotic parasitic diseases and a neglected tropical disease. It affects more than 200 million people globally, with 800 million at risk of infection [1, 2]. In tropical and subtropical areas in Africa and Latin America, particularly in Brazil, schistosomiasis is caused by *S. mansoni* [3] and transmitted by snails of the class Gastropoda, family Planorbidae, genus *Biomphalaria*, especially *Biomphalaria glabrata*, *B. straminea*, *B. pfeifferi*, and *B. tenagophila*, *B. glabrata* is the primary intermediate host for *S. mansoni*, characterized by high infection levels and wide distribution. In contrast, *B. straminea* shows lower infection rates but possesses a greater capacity for resistance to desiccation, better dispersal ability, and higher vagility [4–6], contributing to its success as an invasive species.

*B. straminea* was originally native to Brazil, but its distribution now extends from America to Asia [2, 3]. The first report of *B. straminea* in China was in Hong Kong (HK) in 1974 [7]; its presence in Shenzhen City, Guangdong Province (GDSZ), southern China was subsequently reported in the 1980s [8, 9]. Recent epidemiological surveys indicate that *B. straminea* has expanded across the Pearl River Basin and spread to the surrounding areas, including Dongguan City (GDDG) and Huizhou City (GDHZ) in Guangdong Province [3, 10, 11], suggesting spread from HK rivers. As an invasive species, *B. straminea* exhibits a wide distribution with high density in the rivers of GDSZ [12]. Concurrently, the importation of *S. mansoni*-infected cases into China has risen rapidly [13]. The Greater Bay Area, encompassing GDSZ, GDDG, and HK, is a high-risk zone due to extensive international travel and trade with *S. mansoni*-endemic regions, increasing the likelihood of parasite introduction. Therefore, the risk of schistosomiasis transmission and prevalence in this area requires significant attention.

Controlling the vector and reducing the occurrence and spread of schistosomiasis necessitates understanding the genetic variation and structure of *B. straminea* following its invasion of China and tracking its spread and origins. Despite its decades-long invasive presence in China, the population genetic structure and invasion history of *B. straminea* remain poorly characterized [14, 15]. This knowledge gap has hindered the development of effective molecular tools for source tracing. Molecular markers, such as amplified fragment length polymorphisms (AFLP), restriction fragment length polymorphisms (RFLP), random amplified polymorphic DNA (RAPD), simple sequence repeats (SSR), and single nucleotide polymorphisms (SNPs), provide effective detection methods for tracing sample origin. SNPs, as third-generation molecular markers, have become powerful tools due to their abundance, stability, variation in both coding and noncoding genomic regions, and suitability for high-throughput automation [16–18]. While earlier studies on *B. straminea* utilized markers like 16S ribosomal RNA (rRNA), cytochrome c oxidase subunit I (COI), and internal transcribed spacer (ITS) [14, 15], high-throughput sequencing techniques like restriction-site associated DNA sequencing (RAD-seq) now enable efficient genome-wide SNP discovery and genotyping [19, 20]. After SNP identification via RAD-seq, a highly efficient validation method is the Sequenom MassARRAY iPLEX genotyping platform [21].

In this study, we aim to fill theses gaps by assessing the genetic diversity and population structure of *B. straminea* in the Greater Bay Area (GDSZ, GDDG, and HK) utilizing RAD-seq, identifying and validating geographically specific SNP markers, and predicting the current and future potential distribution of *B. straminea* in China under climate change scenarios using the Maximum Entropy (MaxEnt) model. These findings will provide critical insights for monitoring and controlling this intermediate host of *S. mansoni*.

## Methods

### Field sampling

Based on the previous reports, samples were collected from five sites in GDSZ, two sites in GDDG, and one site in HK between April and August 2017 (Additional file 1). All *Biomphalaria* snails found within the sampling location were collected as extensively as possible and transported alive to the laboratory with water. Furthermore, to screen for *S. mansoni* infection, *Biomphalaria* snails were crushed and examined under a stereoscopic microscope (SZ650, Cnoptec, Chongqing, China) for sporocysts. Live snails were also placed in dechlorinated water and exposed to artificial light for over four hours to stimulate cercariae emission before examination.

### DNA extraction and molecular identification

Following preliminarily morphological diagnosis of *Biomphalaria*, *B. straminea* specimens were selected, and all other species were excluded. Genomic DNA was then extracted from approximately 30 mg muscle tissue from each *B. straminea* individual using the HiPure Tissue DNA Mini Kit (Magen, Guangzhou, China) according to the manufacturer’s recommended protocol. Subsequently, species identification was confirmed by amplifying and sequencing the mitochondrial 16S rRNA and COI genes. PCR was performed in a 50 μl mixture containing approximately 30 ng of DNA template, 1.1 × Golden Star T6 Super PCR Mix (Tsingke, Beijing, China) and 0.4 μmol/L of each primer, using a Bio-Rad PCR C1000 Touch instrument (Bio-Rad, Hercules, CA, USA). The 16S rRNA primer set was: forward 5′-CGCCTGTTTATCAAAAACAT-3′ and reverse 5′-CCGGTCTGAACTCAGATCACGT-3′; the COI primer set was: forward 5′-GGTCAACAAATCATAAAGATATTGG-3′ and reverse 5′-TAAACTTCAGGGTGACCAAAAAATCA-3′ [22, 23]. Negative controls using ultrapure water were included to monitor PCR contamination. The thermal cycling conditions were: initial denaturation at 98 °C for 2 min; 35 cycles of denaturation at 98 °C for 10 s, annealing at 55 °C for 30 s, extension at 72 °C for 15 s, and a final extension at 72 °C for 3 min. Afterwards, the PCR products were purified and inserted into a pClone007 Blunt Simple Vector (Tsingke, Beijing, China), and the plasmids were used to transform *E. coli* DH5ɑ. Sequencing was performed by Tsingke Biotechnology Ltd., Co. (Guangzhou, China). A phylogenetic analysis was conducted by Molecular Evolutionary Genetics Analysis (MEGA) 11 (https://www.megasoftware.net) to confirm species assignment [24].

### Double digest restriction associated DNA (ddRAD) library construction and sequencing

The quality and quantity of *B. straminea* DNA samples were verified using a NanoDrop One Spectrophotometer (Thermo Fisher Scientific, Wilmington, DE, USA) and a Qubit 3.0 Fluorometer (Life Technologies, Carlsbad, CA, USA). Then, ddRAD libraries were prepared following the method of Peterson et al. [25] with minor modifications. Briefly, for each individual, approximately 200 ng of DNA was double digested with the *Eco*RI High-Fidelity restriction enzyme (New England Biolabs, Inc. (NEB), Ipswich, MA, USA) and *Msp*I (NEB). After digestion, *Eco*RI adapter containing individual specific nucleotide barcodes (5 bp) for sample tracking and *Msp*I barcoded adapter were ligated. The ligation products were subsequently pooled in groups of 24 samples with different *Eco*RI adapters and purified using AMPure XP beads (Beckman Coulter, Brea, CA, USA) based on the manufacturer’s instructions. Libraries were then enriched in 25 μl volumes containing each isolated sample, NEBNext^®^ High-Fidelity 2 × PCR Master Mix, PCR primer 1, and PCR Primer 2. Details of the ddRAD nucleotide barcodes are listed in Additional file 2. Subsequently, PCR products for each library were mixed, cleaned with SPRI AMPure XP beads, and pooled in equimolar ratios to create a single library for sequencing. An Agilent 2100 Bioanalyzer (Agilent Technologies, Santa Clara, CA, USA) was used to quantify the molarity of purified PCR product and select fragments sized between 450 and 550 bp. Additionally, ddRAD libraries were quantified by a Qubit 3.0 Fluorometer, and quantitative polymerase chain reactions (qPCR) were performed, concentrations greater than 3 nmol/L were deemed appropriate. Finally, quantified libraries were pooled on the flowcell and then sequenced in different lanes of the HiSeq X-TEN System (Illumina, San Diego, CA, USA) by Novogene Company (Tianjin, China) to obtain 150 bp pair-end (PE) reads.

### SNP calling and filtering

Raw reads were quality-filtered using the NGS QC Tool kit (http://www.nipgr.res.in/ngsqctoolkit.html) [26] to remove adapter-contaminated reads, reads with more than 10% unidentified nucleotides, and paired reads with over 50% low-quality (Phred quality score ≤ 5) bases across the total length [27]. The obtained clean reads were then assigned to each individual based on unambiguous barcodes and the specific recognition sites for *Eco*RI and *Msp*I using Stacks (http://creskolab.uoregon.edu/stacks/) [28], reads lacking the unique barcode and specific sequence were filtered out. Afterwards, effective sequences were individually mapped to the *B. glabrata* reference genome (https://www.ncbi.nlm.nih.gov/assembly/GCF_000457365.1) using Bowtie 2 (http://bowtie-bio.sourceforge.net/bowtie2/index.shtml) [29] with default parameters. The resulting sequence alignment/map (SAM) files for each individual were converted to sorted binary alignment/map (BAM) files using SAMtools (http://samtools.sourceforge.net/) [30]. SNP calling was performed by SAMtools/BCFtools using strict filtering criteria: total depth > 3000, Phred-scaled quality score > 10, read depth per sample ≥ 10, genotype quality ≥ 10, missing data rate ≤ 0.02, and allele frequencies between 0.1 and 0.9. Also, all INDELs (insertions and deletions) were discarded. The retained SNPs were considered high-quality and used for further analysis.

### Population genetic analysis

To evaluate genetic variation and differentiation at each sampling site, the number of different alleles (*Na*), number of effective alleles (*Ne*), expected heterozygosity (*He*), observed heterozygosity (*Ho*), percentage of polymorphic loci, inbreeding coefficient (*Fis*), pairwise genetic distance (*Fst*), and number of effective migrants (*Nm*) were calculated using GenAlEx 6.5 (https://biology.anu.edu.au/research/software/genalex) [31]. Principal component analysis (PCA) was performed by using the R package adegenet [32] based on the high-quality SNPs to compare the genetic relationships among populations. Whereafter, the R package LEA [33] was applied to assign individuals from different populations to clusters using population allele frequencies and ancestry proportions, with cross-validation conducted to select the optimal number of clusters (K ranging from 1 to 10). The existence of genetic clusters was further confirmed using discriminant analysis of principal components (DAPC) with the R package adegenet [34]. To further investigate genetic relatedness among *B. straminea* populations, a minimum spanning network (MSN) was constructed using the R package poppr [35]. Furthermore, a neighbor-joining (NJ) tree was built via MEGA 11 [24] with 1000 bootstrap resamplings for phylogenetic analysis.

### SNP validation by MassARRAY iPLEX assay

To validate the accuracy of SNPs predicted by the NGS data, a subset of eight high-quality SNPs was randomly selected for experimental validation by the iPLEX Gold MALDI-TOF MS system (Sequenom, San Diego, CA, USA) on the same 290 samples. The positions of the selected SNPs, primers used to amplify the flanking sequences, and extension primers designed by Assay Design Suite v2.0 (Sequenom) are listed in Additional file 3. Primers were synthesized by Sangon Biotech (Shanghai, China). Multiplexed PCR was performed in a GeneAmp PCR system 9700 thermal cycler (Applied Biosystems, Waltham, MA, USA) according to the manufacturer’s protocol. The obtained PCR products were then treated with a cocktail of 10 × Shrimp Alkaline Phosphatase (SAP) Buffer, 1.7 U/μl SAP Enzyme and H_2_O in a GeneAmp PCR system 9700 thermal cycler with dephosphorylation, followed by inactivation of SAP enzyme. Afterwards, single-base extension (SBE) reactions were conducted with the SAP treated PCR products and iPLEX Gold extension reaction cocktail in a GeneAmp PCR system 9700 thermal cycler. All SBE products were dispensed onto a 384-format SpectroCHIP (Sequenom) using the MassARRAY Nanodispenser RS 1000 (Sequenom), and MALDI-TOF MS analysis was subsequently performed with all samples typed in duplicate. Data acquisition was automated using SpectroAcquire (Sequenom), and data analysis was performed with MassARRAY Typer 4.0 software (Sequenom). The genotyping results of SNPs from the SpectroCHIP and NGS datasets were compared.

### Prediction of potential distribution using MaxEnt model

The occurrence point data of *B. straminea* in China were obtained from the Global Biodiversity Information Facility (GBIF, https://www.gbif.org), literature, and this study. After removing duplicates, 79 unique distribution points were saved as a comma-delimited (CSV) file for further analysis (Additional file 4). Nineteen bioclimatic variables (Bio 1–Bio 19) with a spatial resolution of 10 arc-minutes were sourced from the WorldClim database (https://worldclim.org) and listed in Additional file 5. Then, to predict future distributions of the species, scenarios in the 2030 s under different greenhouse gas emission levels (SSP1-2.6, SSP2-4.5, and SSP5-8.5) from the Moderate Resolution National Climate Center (Beijing) Climate System Model (BCC-CSM2-MR) of the Coupled Model Intercomparison Project Phase 6 (CMIP6) were used. These environmental data were converted from TIF to ASCII format using ArcGIS v10.7 (ESRI Inc., Redlands, CA, USA).

MaxEnt v3.4.3 (https://biodiversityinformatics.amnh.org/opensource/maxent/) [36] was employed to predict suitable habitats for *B. straminea*. The distribution points and all environmental variables were imported into the model, with 25% of the distribution data used for testing and the remaining 75% for training. The maximum number of background points was specified as 10,000, the iteration was repeated 10 times using a bootstrap, and other parameters were set to default values. Model performance was evaluated using the area under the curve (AUC), a value greater than 0.9 indicates high performance. Also, a jackknife test was used to measure variable importance in affecting the distribution of *B. straminea*. Finally, MaxEnt model predictions were imported into ArcGIS v10.7 for better visualization and reclassification. Using the Jenks’ natural break method, habitat suitability was classified into four categories: unsuitable, lowly suitable, moderately suitable, and highly suitable.

## Results

### Schistosome infection status and molecular characterization of *B. straminea*

We collected 290 *B. straminea* individuals containing 171 samples from GDSZ, 65 from GDDG, and 54 from HK. No sporocyst or cercaria of *S. mansoni* were found in any collected snails upon examination under a stereoscopic microscope. Moreover, phylogenetic analysis of *Biomphalaria* based on the sequences obtained in this study and reference sequences with high similarity obtained from GenBank for the COI gene showed a distinct clade for all *B. straminea* individuals, strongly supported by bootstrap values of 94–100%, confirming that the collected species were *B. straminea* (Additional file 6).

### SNP discovery and distribution patterns

**Fig. 1:**
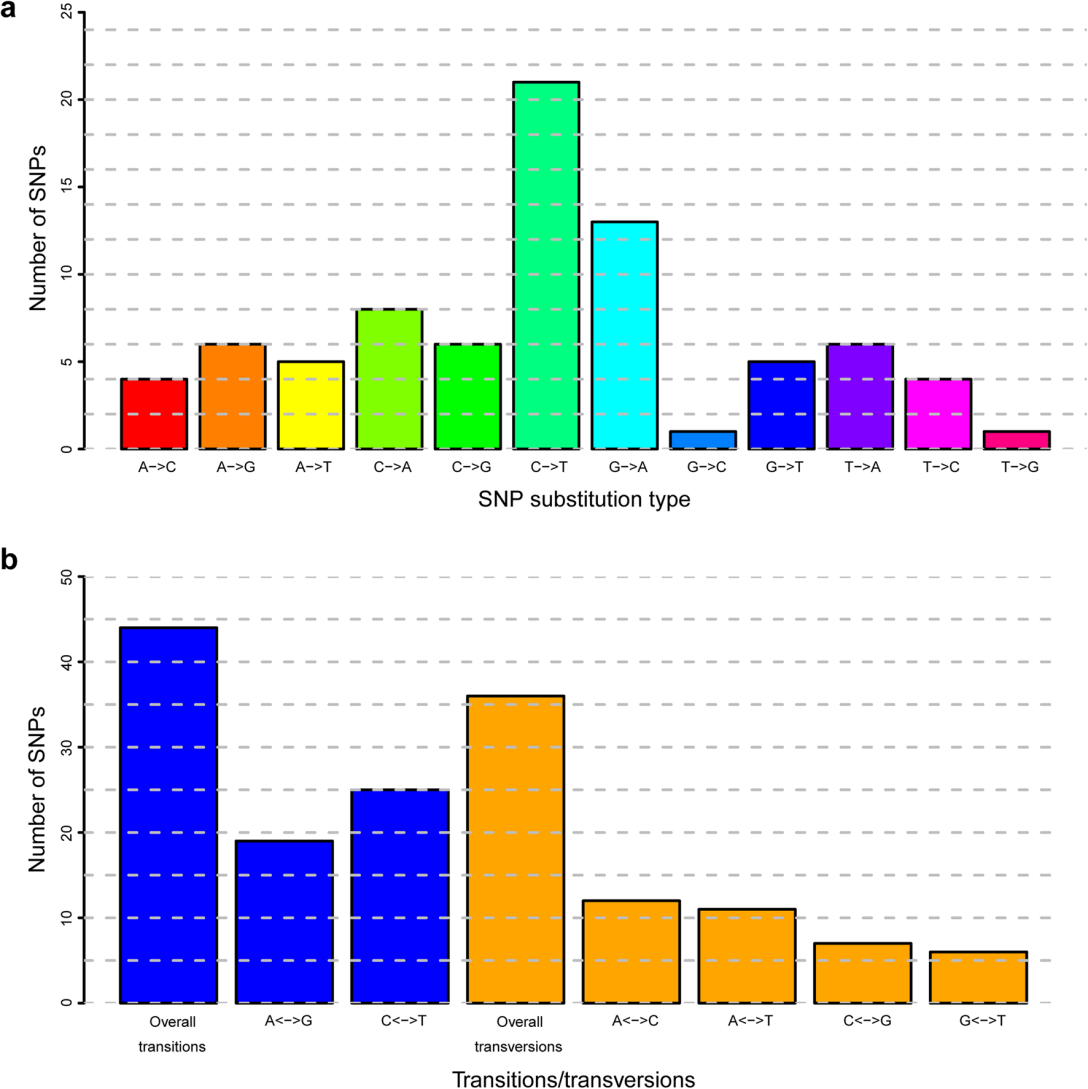
Distribution patterns of SNPs discovered in the populations of *Biomphalaria straminea*. **a** The number of SNP substitutions. **b** The count of SNP transitions and transversions. *SNP* single nucleotide polymorphism

Using *Eco*RI-*Msp*I digestion, we successfully constructed ddRAD libraries containing 290 samples. High-throughput sequencing yielded over 1 billion PE reads, with approximately 600 million clean reads retained after quality control (Additional file 7). As the *B. straminea* genome was unavailable at the time, high-quality reads were aligned against the *B. glabrata* genome. Unexpectedly, only approximately 20% of reads aligned to the reference genome. From these aligned reads, 37,797 putative SNPs were identified, but only 80 high-quality SNPs were retained for downstream genetic diversity and population structure analysis after strict filtering. Detailed SNP information is provided in Additional file 8. Analysis of distribution patterns among the 80 high-quality SNPs showed that the most common variation type was C/T (21), followed by G/A (13) and C/A (8), whereas G/C (1) and T/G (1) had the lowest proportion (Fig. 1a). In total, 44 SNPs were identified as transitions and 36 as transversions (Fig. 1b), yielding a transition/transversion ratio of 1.22. This suggests a higher frequency of purine to purine or pyrimidine to pyrimidine substitutions compared to purine-pyrimidine substitutions in *B. straminea*.

### Population genetic diversity and differentiation

According to the population genetic data listed in Table 1, *Na* per population ranged from 1.78 in HK to 1.99 in GDSZ, whereas *Ne* per population ranged from 1.40 in HK to 1.45 in GDDG. *He* and *Ho* also varied among sites, with the highest values observed in *B. straminea* from GDDG (*He* = 0.28, *Ho* = 0.37). However, samples from GDSZ presented the highest percentage of polymorphic loci. In conclusion, population genetic characteristics revealed that *B. straminea* from GDSZ had higher genetic diversity than those from other locations. Furthermore, *Ho* in all populations was higher than *He*, resulting in negative *Fis* values, indicating that outbreeding has dominated their recent genetic history.

**Table 1:** Population genetic data for the *Biomphalaria straminea* populations

Population	*N*	*Na*	*Ne*	*He*	*Ho*	%P	*Fis*
GDSZ	171	1.99	1.43	0.27	0.35	0.99	− 0.28
GDDG	65	1.95	1.45	0.28	0.37	0.95	− 0.32
HK	54	1.78	1.40	0.24	0.34	0.78	− 0.46
Total	290	1.90	1.43	0.26	0.35	0.90	− 0.35

*GDSZ* Shenzhen City, Guangdong Province, China; *GDDG* Dongguan City, Guangdong Province, China; *HK* Hong Kong, China; *N* number of individuals; *Na* number of different alleles; *Ne* number of effective alleles; *He* expected heterozygosity; *Ho* observed heterozygosity; *%P* percentage of polymorphic loci; *Fis* inbreeding coefficient

The degree of genetic differentiation among the three populations was estimated by calculating *Fst* and *Nm*. The *Fst* value between GDDG and HK was the highest (*Fst* = 0.052), while the smallest value was observed between GDDG and GDSZ (*Fst* = 0.016), and the divergence between GDSZ and HK was intermediate (*Fst* = 0.044) (Table 2). Furthermore, *Nm* values ranged from 4.558 to 15.375 (Table 2), indicating frequent gene flow among the three populations. These results demonstrate low genetic differentiation between population pairs of *B. straminea* and a closer genetic relationship between GDDG and GDSZ.

**Table 2:** Pairwise genetic distance (*Fst*) (below diagonal) and number of effective migrants (*Nm*) (above diagonal) among the populations of *Biomphalaria straminea*

Population	GDSZ	GDDG	HK
GDSZ	–	15.375	5.432
GDDG	0.016	–	4.558
HK	0.044	0.052	–

*GDSZ* Shenzhen City, Guangdong Province, China; *GDDG* Dongguan City, Guangdong Province, China; *HK* Hong Kong, China

### Population structure, relationship, and phylogenetic analysis

**Fig. 2:**
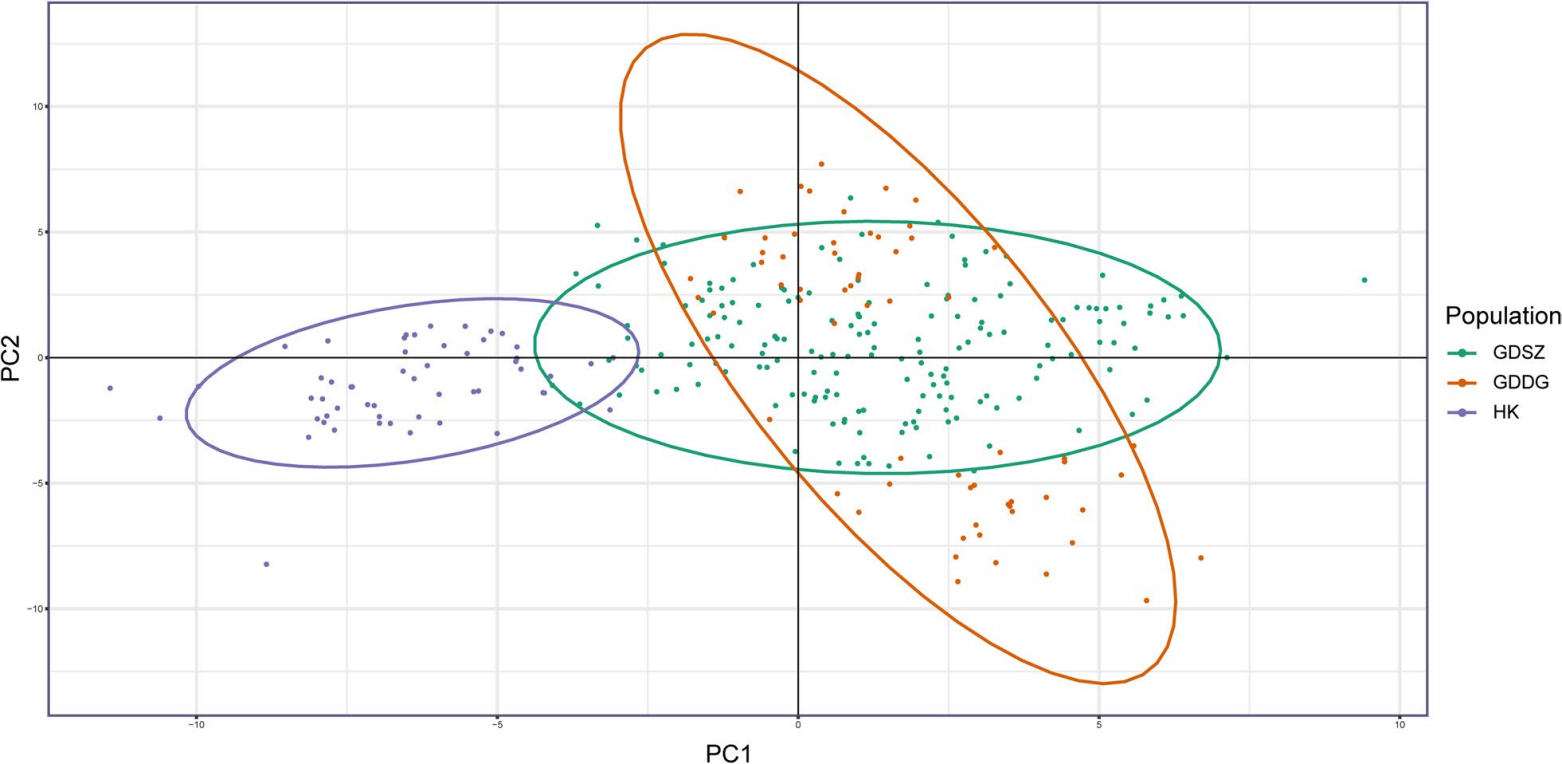
Principal component analysis (PCA) of *Biomphalaria straminea* populations. Each individual is represented by one point, with its color corresponding to the location from which the individual was collected. *GDSZ* Shenzhen City, Guangdong Province, China; *GDDG* Dongguan City, Guangdong Province, China; *HK* Hong Kong, China

PCA was applied to visualize individual relationships within and between populations using 80 high-quality SNPs generated from 290 *B. straminea* samples. Obviously, samples from HK formed a tight cluster, while individuals from GDSZ and GDDG clustered together relatively, indicating they were genetically closer (Fig. 2). Principal component (PC) 1 and PC 2 accounted for only 5.24% and 3.57% of the total variation, respectively, a common feature in genetic studies of populations with high gene flow and low differentiation. PC 1 clearly distinguished the HK population from the others, while PC 2 separated *B. straminea* from GDSZ and GDDG.

**Fig. 3:**
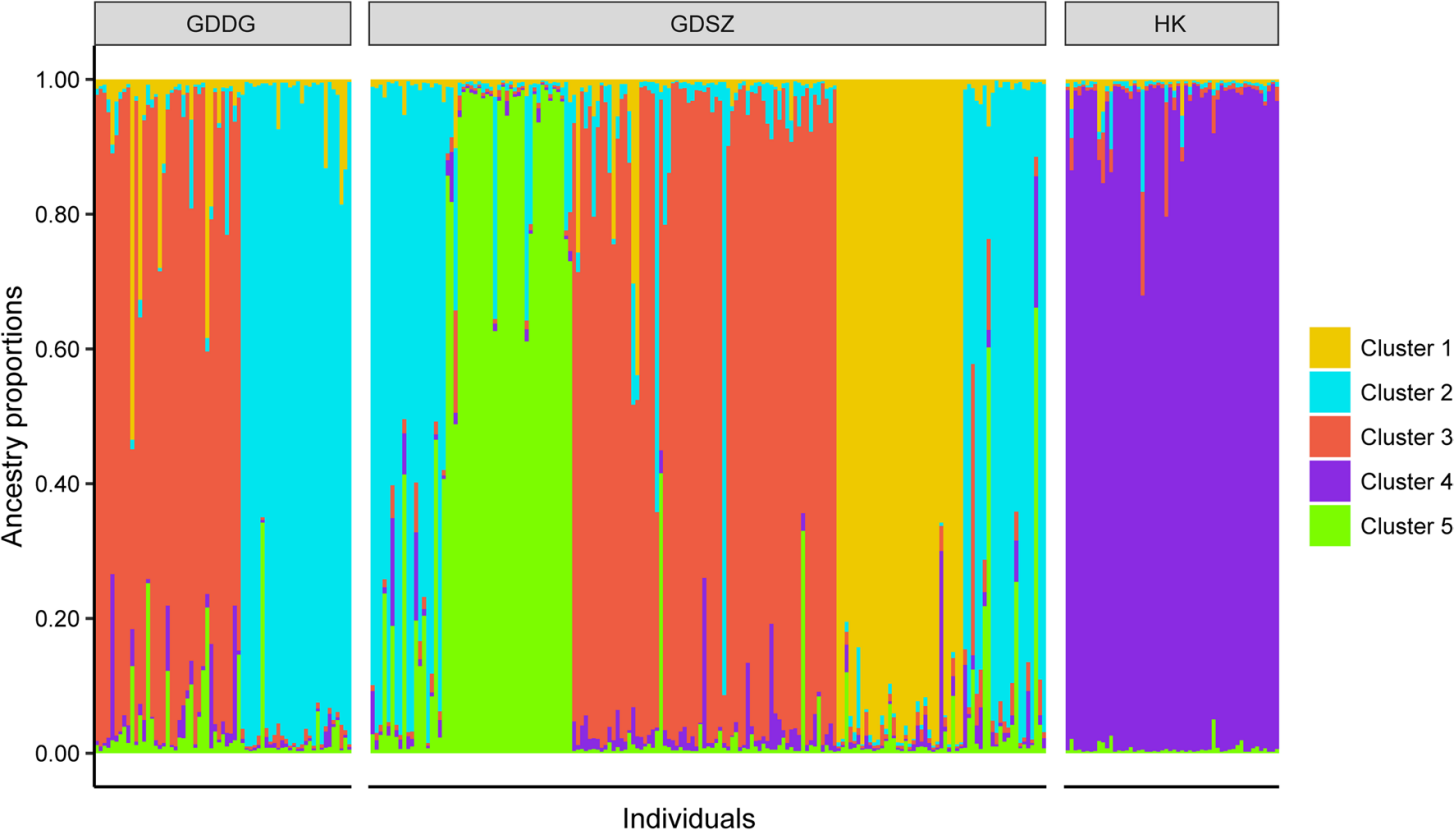
Structure analysis among the populations of *Biomphalaria straminea*. Each vertical bar indicates an individual, and different colors represent the proportion of genomic components in an individual derived from different genetic clusters. *GDSZ* Shenzhen City, Guangdong Province, China; *GDDG* Dongguan City, Guangdong Province, China; *HK* Hong Kong, China

Structure analysis, which expanded on the PCA results and revealed potential admixture among populations, suggested that K = 5 was the optimal number of clusters. Thus, all *B. straminea* populations could be divided into five subgroups. Individuals from GDDG and some from GDSZ shared the same genetic clusters (Cluster 2 and 3), while other individuals from GDSZ showed unique genetic clusters (Cluster 1 and 5), and the HK population formed a separate isolated cluster (Cluster 4) (Fig. 3). Furthermore, varying degrees of admixture were observed in all populations, especially in individuals from GDSZ and GDDG, further confirming the high genetic polymorphism in these two populations.

**Fig. 4:**
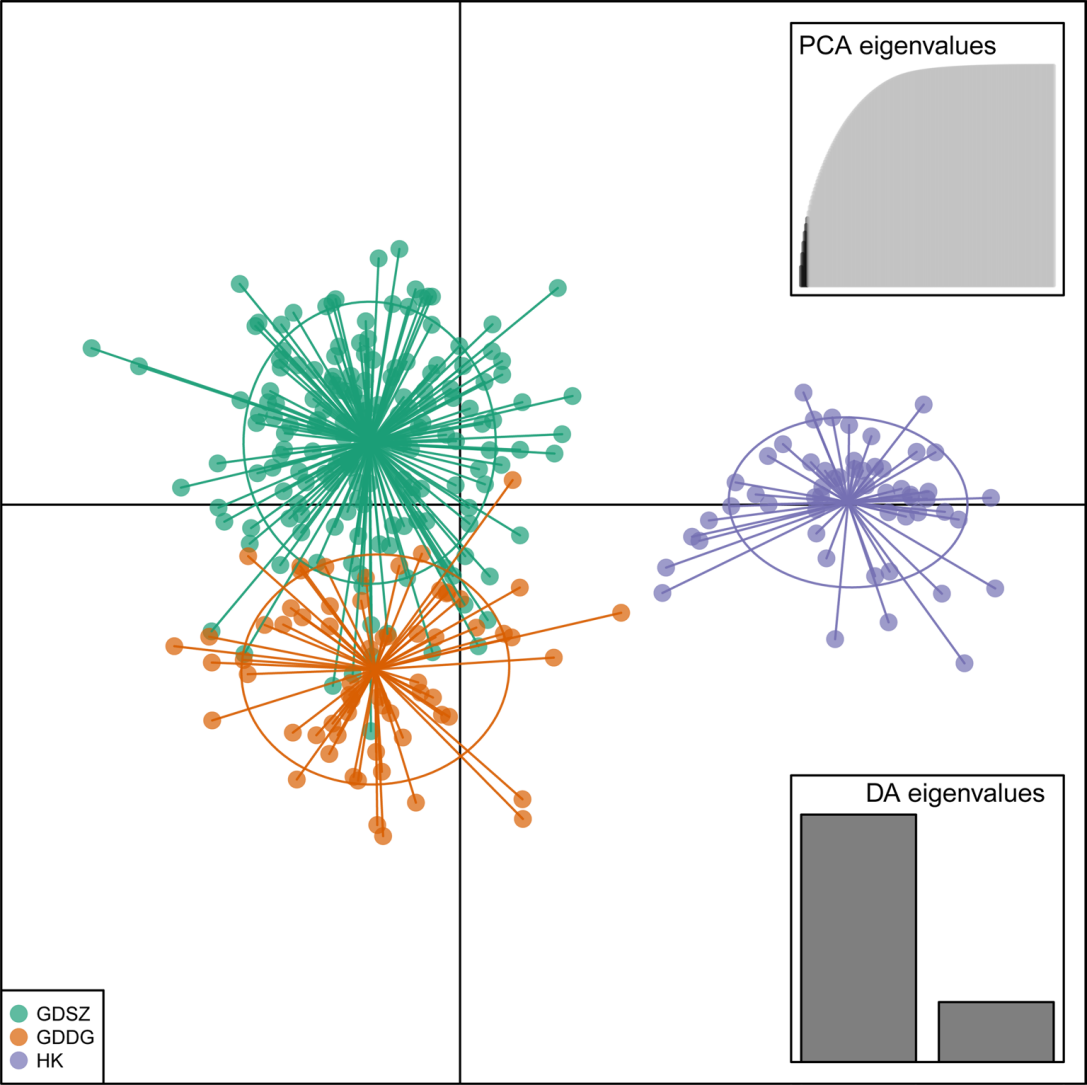
Discriminant analysis of principal components (DAPC) for *Biomphalaria straminea* populations. Each individual is a dot, and each population is exhibited by a different color. *GDSZ* Shenzhen City, Guangdong Province, China; *GDDG* Dongguan City, Guangdong Province, China; *HK* Hong Kong, China

We then employed DAPC to validate the population structure mentioned above. However, the DAPC results indicated that the *B. straminea* populations should be divided into three groups (Fig. 4), revealing that although high genetic polymorphism existed within the populations, genetic differentiation among different sampling sites within the same location was insufficient to subdivide them further. The DAPC results also showed that individuals from HK were distinct from those of other populations, supporting a higher degree of genetic differentiation. In contrast, samples from GDSZ and GDDG partially overlapped, indicating a closer genetic relationship. Overall, the DAPC and PCA results were quite similar.

**Fig. 5:**
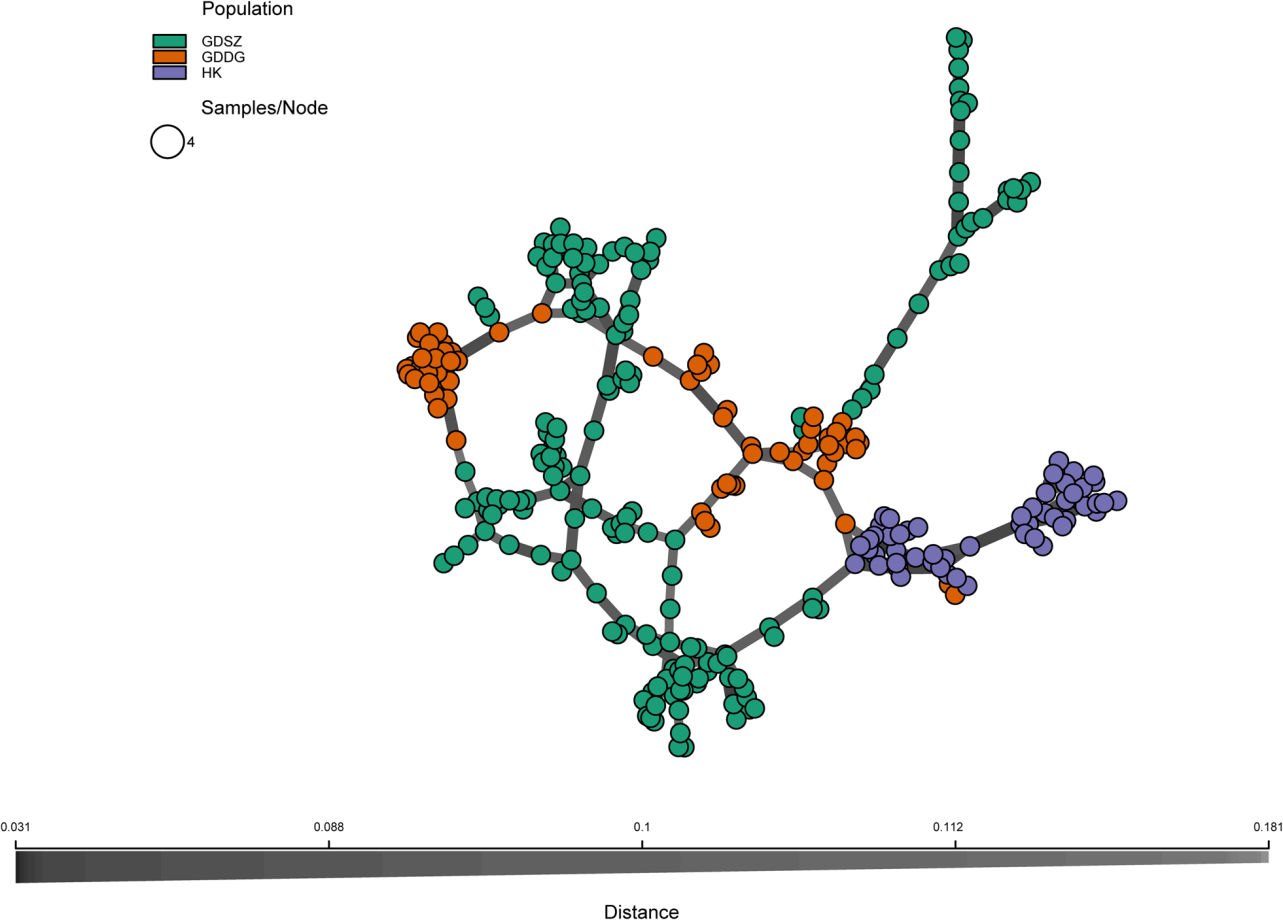
Minimum spanning network (MSN) of *Biomphalaria straminea* individuals. Each individual is a node, and the edge indicates the genetic distance. Different colored nodes represent different locations from which the samples were collected. *GDSZ* Shenzhen City, Guangdong Province, China; *GDDG* Dongguan City, Guangdong Province, China; *HK* Hong Kong, China

The MSN exhibited in Fig. 5 revealed that *B. straminea* populations were differentiated geographically. Populations from HK were genetically linked, and some samples from GDDG and GDSZ were closely related to HK samples, suggesting the former might originate from HK. Nevertheless, all the samples were closely related in the network, further confirming the low genetic differentiation among *B. straminea* populations.

**Fig. 6:**
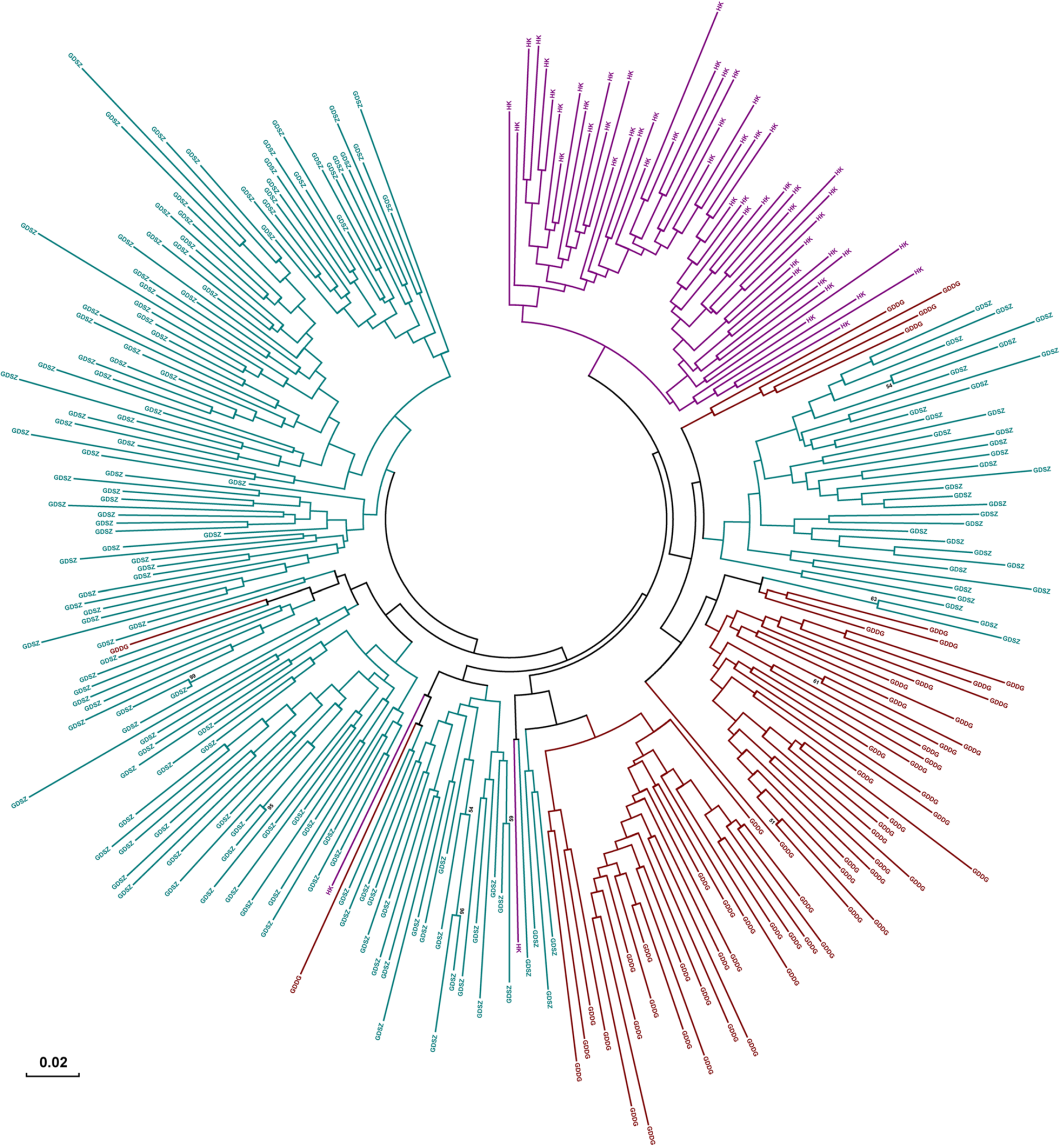
Phylogenetic tree of *Biomphalaria straminea* populations. The tree was constructed using the neighbor-joining method based on the Kimura 2-parameter model. Bootstrapping values are exhibited on the branches, and the scale bar indicates genetic distance. *GDSZ* Shenzhen City, Guangdong Province, China; *GDDG* Dongguan City, Guangdong Province, China; *HK* Hong Kong, China

Finally, a NJ tree was constructed using the high-quality SNPs to infer evolutionary relationships among populations (Fig. 6). The tree exhibits two major genetic clusters: one clade includes some samples from GDSZ, and the other contains the remaining samples. Consistent with the MSN results, some individuals from GDDG and GDSZ, as well as individuals from HK, were more closely related in the NJ tree, indicating they might share the same origin. Furthermore, we observed that all samples clustered mainly by region, revealing that the selected SNPs could distinguish *B. straminea* from different locations.

### Validation of geographically specific SNP markers

**Fig. 7:**
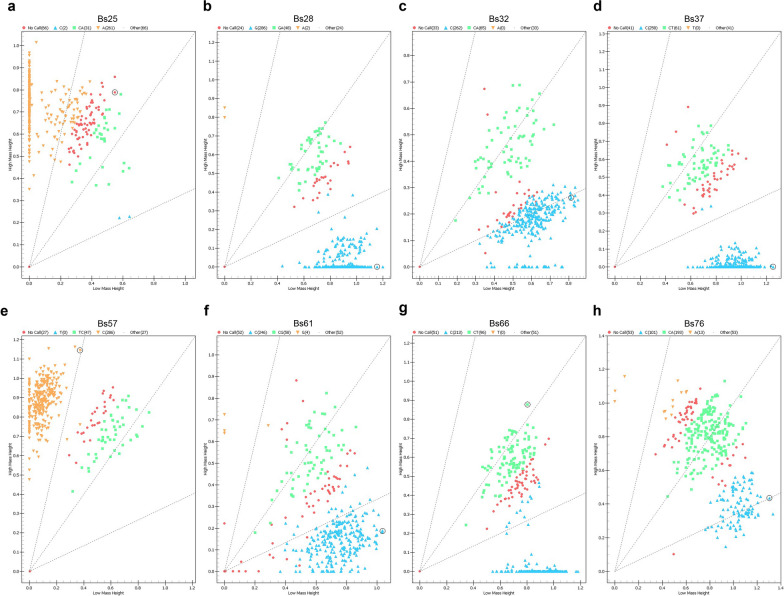
Distribution of genotypes from eight SNP loci of *Biomphalaria straminea*. **a** Bs25. **b** Bs28. **c** Bs32. **d** Bs37. **e** Bs57. **f** Bs61. **g** Bs66. **h** Bs76. *SNP* single nucleotide polymorphism

We employed the Sequenom MassARRAY iPLEX assay to confirm the predictability of eight high-quality SNPs on the same set of 290 samples. The distribution of genotypes for various SNP loci is shown in Fig. 7 (‘no call’ indicates no extension product was detected). Genotyping results for all samples are summarized in Additional file 9, and mass spectra of the genotyping results from different SNP loci of *B. straminea* using the iPLEX assay are shown in Additional file 10. As expected, genotyping results from the iPLEX assay were largely concordant with those from ddRAD-seq.

Furthermore, we found that three of eight high-quality SNPs displayed distinct geographical population specificity (Table 3) and could be used as geographically specific SNP markers for tracing *B. straminea* samples. Mutated alleles of the Bs25 locus were only observed in samples from GDSZ, and samples from GDSZ and GDDG both showed different levels of mutation at the Bs37 locus. Therefore, examining these three SNP loci individually: if a mutation exists at the Bs76 locus, the collected *B. straminea* might be from GDSZ, GDDG or HK; if a mutation is found at the Bs37 locus, the snail might be from GDSZ or GDDG; if the collected *B. straminea* is from GDSZ, a mutation at the Bs25 locus would be expected. In addition, if we test the three SNP loci sequentially, assigning the wild-type allele as 0 and the mutant allele as 1, a result of 111 would suggest *B. straminea* from GDSZ, 110 would suggest GDDG origin, and 100 would suggest the sample might be from HK.

**Table 3:** Mutation frequency of three SNP loci among the populations of *Biomphalaria straminea*

Population	Bs25	Bs37	Bs76
GDSZ	11.51%	7.19%	27.67%
GDDG	–	30.00%	47.37%
HK	–	–	56.41%

*GDSZ* Shenzhen City, Guangdong Province, China; *GDDG* Dongguan City, Guangdong Province, China; *HK* Hong Kong, China

### Current and future potential distribution of *B. straminea*

The optimized MaxEnt model achieved an AUC value of 0.984 (Additional file 11a), demonstrating excellent performance in predicting suitable habitats for *B. straminea*. Based on percentage contributions and Jackknife test results, four key environmental variables were identified: annual mean temperature (Bio 1), isothermality (Bio 3), mean temperature of wettest quarter (Bio 8), and annual precipitation (Bio 12). Among these, Bio 1 (68.4%) and Bio 8 (15.4%) accounted for over 80% of the total contribution. Also, the Jackknife test results showed that these two variables had longer blue bars, indicating their greater importance for potential distribution (Additional file 11b).

**Fig. 8:**
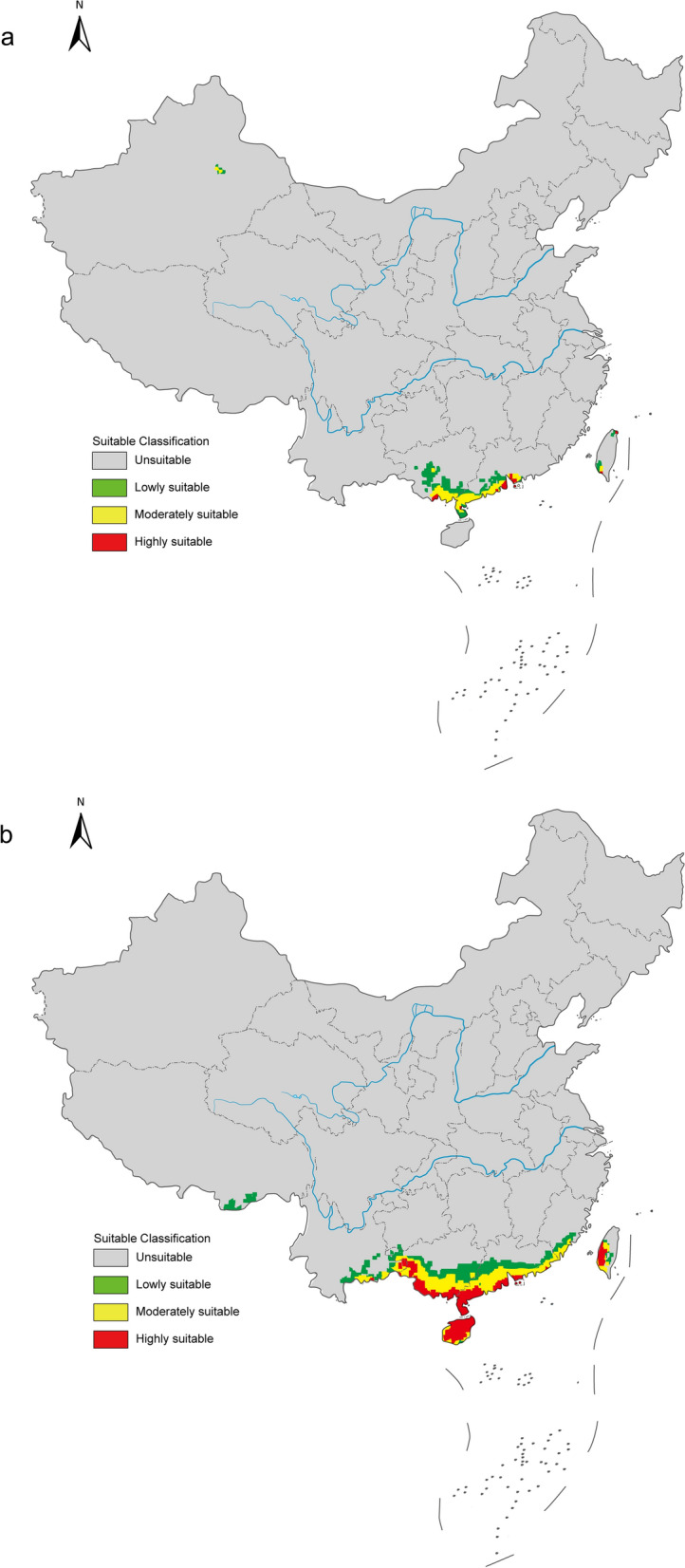

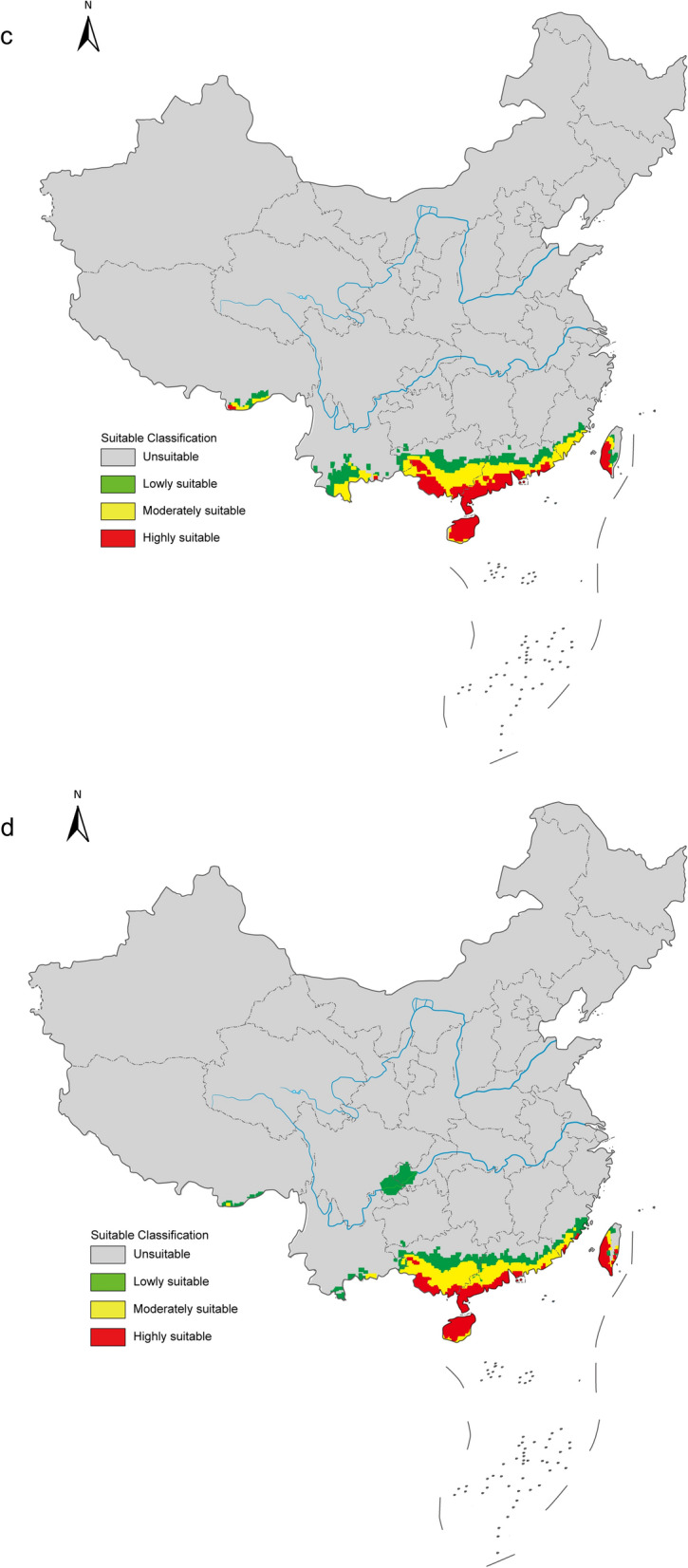
**a** Potential distribution of *Biomphalaria straminea* in China under current climate scenario by the MaxEnt model. Future distribution of *Biomphalaria straminea* in China under the SSP1-2.6 (**b**), SSP2-4.5 (**c**), and SSP5-8.5 (**d**) emission scenarios in the 2030 s by the MaxEnt model. Map approval number: GS(2025)5958

Under current climatic conditions in China, suitable habitats for *B. straminea* were distributed in southern Guangdong Province, southern Guangxi Zhuang Autonomous Region, Taiwan Province, and small areas of Xinjiang Uygur Autonomous Region and Xizang Autonomous Region. Highly suitable habitats were found only in the first three regions, highlighting potential environmental limitations on the establishment and survival of *B. straminea* (Fig. 8a).

As shown in Fig. 8b and c, notable increases in habitat suitability for *B. straminea* were observed in the 2030 s under the SSP1-2.6 and SSP2-4.5 scenarios. Areas such as Hainan Province, southern Yunnan Province, southwestern Guizhou Province, and southeastern Fujian Province, which are unsuitable under current environmental conditions, became suitable. Furthermore, Hainan Province and a small area of Yunnan Province became highly suitable under the SSP1-2.6 scenario, whereas small areas of Fujian Province and Xizang Autonomous Region also became highly suitable under the SSP2-4.5 scenario. Interestingly, Xizang was no longer highly suitable under the SSP5-8.5 scenario, but some areas of Sichuan Province and Chongqing Municipality transited from unsuitable to lowly suitable habitats (Fig. 8d). In conclusion, compared to current climatic conditions, suitable areas for *B. straminea* are projected to increase under all three future greenhouse gas emission scenarios, suggesting that warming has a positive impact on its distribution.

## Discussion

### Risk of *S. mansoni* in China

*B. straminea* is an invasive freshwater snail with strong reproductive capability, drought tolerance, and significant potential for peripheral expansion and intercontinental dispersal [1, 37]. Although China is not currently endemic for *S. mansoni*, the transmission of human schistosomiasis depends on the existence and geographical distribution of its intermediate hosts [38, 39]; therefore, the invasion of *B. straminea* in China demands close attention. On the other hand, in the context of globalization, rapid development of international trade and tourism have undoubtedly increased China’s risk of schistosomiasis. Many Africans with unknown *S. mansoni* infection status have immigrated to Guangzhou and nearby cities [40], and imported *S. mansoni*-infected cases have been observed in China since the 1990 s [41]. Moreover, global warming and climate change may accelerate the diffusion of *B. straminea* due to its excellent adaptability to different environmental conditions [42]. Besides, some *B. straminea* habitats exist in rivers flowing through communities and parks in cities, where citizens, especially children, usually play in the water during summer. It is concerning that if *S. mansoni* cercariae were present in these rivers, people could become infected during water contact, leading to serious public health problems. Fortunately, detection of schistosome larvae in invasive *B. straminea* in China has remained negative to date, and the current distribution of *B. straminea* is confined to Guangdong Province and HK [40]. However, given the relatively low sample size, the possibility of undetected schistosome infection cannot be ruled out. Therefore, elucidating the population structure and genetic diversity of *B. straminea* and discovering geographically specific SNP markers for tracing its geographic origin are particularly important for schistosomiasis prevention and control.

### Population structure and genetic diversity of *B. straminea* in China

Although the spatial distribution of *B. straminea* in China is relatively clear [43], genetic research on its various populations remains limited. Leveraging ddRAD-seq, this study selected 80 high-quality SNP markers to evaluate the population structure and genetic diversity of *B. straminea* in China in a cost-effective manner. Although 80 SNPs is a modest number, they proved sufficient for our analysis and revealed consistent and significant population patterns. Population genetic data indicated that individuals from GDSZ had higher genetic variation than those from other locations, suggesting stronger adaptive capacity, potentially enabling better adjustment to environmental changes [44] and possibly posing a great future public health threat. The increased genetic diversity in GDSZ may also result from multiple source introductions of *B. straminea*. Moreover, the total observed heterozygosity (*Ho* = 0.35) was higher than the expected heterozygosity (*He* = 0.26), revealing a low degree of inbreeding in *B. straminea* populations. The negative *Fis* values indicate outbreeding, which likely enhances the invasive potential of *B. straminea* by maintaining genetic diversity and reducing inbreeding depression [45]. Generally, an *Fst* value lower than 0.05 indicates minimal genetic differentiation, while a value higher than 0.25 indicates great differentiation [46]. Similarly, *Nm* < 1 suggests low gene flow, whereas *Nm* > 4 indicates very high gene flow [47]. In this study, low degree of differentiations and very high gene flows were observed among the tested *B. straminea* populations, suggesting a common origin and ongoing dispersal, likely facilitated by human activities and interconnected waterways. Our results suggested that the presence of *B. straminea* in GDSZ and GDDG resulted from various invasion pathways from HK, including accidental transport and spread through river or water systems [14, 48]. These findings align with recent studies in Brazil and China that also report high connectivity among invasive *B. straminea* populations [15, 49]. Interestingly, low intra-population genetic diversity and high inter-population genetic diversity were observed in *B. pfeifferi* populations from Africa, suggesting low gene flow and high inbreeding levels [50, 51].

To reveal the genetic structures and relationships among *B. straminea* populations, we applied multiple methods, such as PCA, DAPC, population structure analysis, and phylogenetic analysis. The NJ method was chosen for phylogenetic analysis due to its computational efficiency and proven suitability for visualizing genetic relationships based on SNP datasets. In summary, the results consistently demonstrated that the *B. straminea* populations in GDSZ, GDDG, and HK were genetically similar, with individuals from GDDG genetically closer to those from GDSZ, sharing similar genetic backgrounds. Furthermore, structure analysis showed admixture among these populations with varying proportions of genetic components. Relatively speaking, HK populations were purer with less admixture, while GDSZ and GDDG populations exhibited extensive admixture. It is plausible that although the geographic origin of GDSZ individuals was HK, they developed relatively unique genetic structural characteristics after establishing in GDSZ, leading to divergence from the HK population. Subsequently, some individuals from GDSZ invaded GDDG and established the GDDG population. However, migration, mutation, genetic drift, habit heterogeneity, and selection all influence genetic differentiation between populations [52].

### Application of geographically specific SNP markers for *B. straminea*

Although ddRAD-seq yields promising results, the procedure is more tedious compared to the iPLEX assay. The latter is more convenient and cost-effective as all reactions are performed in the same plate, and analyses are automated by specific software [53], making it more suitable for large-scale screening. Our results revealed that the iPLEX assay was ideal for high-throughput genotyping of *B. straminea*. Some failed calls inevitably occurred in the iPLEX assay, possibly due to lower DNA loads or sequence variation in the primer targets. However, overall concordance with ddRAD-seq was high, and the issue of a few failed calls could be resolved by complementing with other methods, such as NGS [54].

To the best of our knowledge, no previous research has investigated population genetic diversity and differentiation along with the population structures and relationships among *B. straminea* populations to this extent. Moreover, the geographically specific SNP markers discovered in the present study were proven useful for tracing the geographic origin of *B. straminea*. Thus, our study provides valuable information for monitoring the intermediate host *B. straminea* and preventing the spread of schistosomiasis. Nevertheless, there is an urgent need to optimize the SNP chip developed here to predict the geographic origin of *B. straminea* with higher accuracy, even among closely related individuals. We anticipate that an optimized SNP chip will become commercially available in the near future.

### Invasion risk assessment for *B. straminea*

Using the MaxEnt model, we predicted suitable habitats for *B. straminea* under current environmental conditions and future climate change scenarios. Although potential suitable areas in China are currently constrained to tropical and subtropical regions, with high suitability habit generally shifting southward, the strong adaptability of *B. straminea* to various climate changes should not be underestimated. The prediction of Xizang Autonomous Region, Sichuan Province, and Chongqing Municipality as suitable habitats in the 2030 s indicates that global climate change could cause an expansion of suitable habitats towards higher latitudes. More importantly, the high invasion risk of *B. straminea* in Hainan Province, Guangxi Zhuang Autonomous Region, and Taiwan Province warrants attention, and more actions should be taken to monitor and block the spread of this species. Furthermore, more suitable areas were observed under higher carbon dioxide emission levels, suggesting that not only high temperature but also high carbon dioxide emissions favor the distribution of *B. straminea*. These results are consistent with findings for *Pomacea canaliculata*, another invasive alien species in China, under warming scenarios [55].

Although the MaxEnt model has some limitations, our findings underscore the potential consequences of environmental changes on the distribution and spread of *B. straminea*. Future climate conditions appear favorable for the survival of this species. These research results can assist the government in implementing more effective and specific measures to limit the invasion of *B. straminea*.

### Limitations

This study has several limitations. First, the use of a divergent reference genome of *B. glabrata*, which resulted in a low alignment rate (~ 20%) and potentially reduced SNP discovery. This specific genome version was later suppressed by National Center for Biotechnology Information (NCBI) due to contamination concerns, but was the best available at the time of analysis. Future studies would benefit from a *B. straminea*-specific genome.

Second, due to a lack of samples from GDHZ, where the sample size was too small for population structure analysis, further investigation with increased sampling from wider locations is important for obtaining a clearer invasion history.

Third, while the samples collected in 2017 provide a crucial baseline, they may not reflect recent genetic changes, future studies should include recent samples to monitor temporal genetic dynamics. Furthermore, samples were collected across multiple months, but temporal variation was not analyzed and should be considered in future studies.

Finally, the MaxEnt model predictions may be biased by limited occurrence data and do not account for other environmental factors, biotic interactions or human-mediated dispersal.

## Conclusions

This study provides the first comprehensive analysis of the population genetics and invasion risk of *B. straminea* in China using genome-wide SNPs and ecological modeling. Our results indicated that populations in GDSZ, GDDG, and HK were genetically similar, with a closer genetic relationship between GDDG and GDSZ. Genetic differentiation among *B. straminea* populations was low, and the GDSZ population exhibited higher diversity than the others. Therefore, our results supported the hypothesis that populations in GDSZ and GDDG resulted from various invasion pathways from HK. We suggested that individuals from GDSZ developed relatively unique genetic structural characteristics after establishment, potentially enhancing their adaptability to environmental changes. Population structure, PCA, DAPC, MSN, and NJ tree all identified three major groups, indicating that the SNPs can be utilized for molecular tracing. Encouragingly, we identified and validated three SNPs with distinct geographical specificity that can serve as markers for rapidly predicting the geographic origin of *B. straminea*. The MaxEnt model predicted an expansion of suitable habitats for *B. straminea* in China under future climate conditions, primarily influenced by temperature and carbon dioxide emissions. High invasion risk in Hainan Province, Guangxi Zhuang Autonomous Region, and Taiwan Province requires attention. These findings are significant from a public health perspective, as *B. straminea*, an intermediate host of *S. mansoni*, has invaded and spread in southern China. Furthermore, globalization and the Belt and Road Initiative have increased the number of *S. mansoni*-infected cases in China. Thus, a better understanding of the population structure and genetic diversity of *B. straminea*, coupled with the application of geographically specific SNP markers to trace its geographic source, will be beneficial for preventing schistosomiasis transmission.

## Supplementary Information

Supplementary material 1. Sampling locations of *Biomphalaria straminea* populations in China.

Supplementary material 2. The ddRAD nucleotide barcodes used in this study

Supplementary material 3. The primers used in the iPLEX assay

Supplementary material 4. Distribution points of *Biomphalaria straminea* used in the MaxEnt model.

Supplementary material 5. Description of 19 environmental variables used in the MaxEnt model to predict the potential distribution of *Biomphalaria straminea. *

Supplementary material 6. Phylogenetic tree of COI gene among *Biomphalaria*. The tree was constructed by using the Maximum Likelihood method based on the Jones-Taylor-Thornton (JTT) model. The relative values (%) on branches are based on 1000 bootstrap resamplings. *Planorbella trivolvis* was used to form the outgroup. *Biomphalaria straminea* identified in the present study were shown in the square frame.

Supplementary material 7. Quality evaluation of reads obtained from ddRAD-seq

Supplementary material 8. Detailed information of the 80 high-quality SNPs

Supplementary material 9. Genotyping results obtained from high-throughput sequencing and iPLEX assay of all 290 *Biomphalaria straminea* samples.

Supplementary material 10. Mass spectra of different genotyping results acquired from eight SNP loci of *Biomphalaria straminea*. **a** Bs25. **b** Bs28. **c** Bs32. **d** Bs37. **e** Bs57. **f** Bs61. **g** Bs66. **h** Bs76.

Supplementary material 11. **a** Receiver operating characteristic (ROC) curve for the dataset of *Biomphalaria straminea* generated by the MaxEnt model. **b** Jackknife test of regularized training gain for the MaxEnt model of *Biomphalaria straminea *distribution.

## Data Availability

The raw sequence data were deposited in the NCBI Sequence Read Archive (SRA) under project number PRJNA863069.

## Abbreviations

AFLP: Amplified fragment length polymorphisms
AUC: Area under the curve
BAM: Binary alignment/map
COI: Cytochrome oxidase subunit I
DAPC: Discriminant analysis of principal components
ddRAD: Double digest restriction associated DNA
GBIF: Global Biodiversity Information Facility
GDDG: Dongguan City, Guangdong Province, China
GDHZ: Huizhou City, Guangdong Province, China
GDSZ: Shenzhen City, Guangdong Province, China
HK: Hong Kong, China
INDEL: Insertion and deletion
ITS: Internal transcribed spacer
MALDI-TOF: Matrix-assisted laser desorption ionization time-of-flight
MaxEnt: Maximum Entropy
MEGA: Molecular Evolutionary Genetics Analysis
MS: Mass spectrometry
MSN: Minimum spanning network
NCBI: National Center for Biotechnology Information
NGS: Next generation sequencing
NJ: Neighbor-joining
PC: Principal component
PCA: Principal component analysis
PCR: Polymerase chain reactions
qPCR: Quantitative polymerase chain reactions
RAD-seq: Restriction-site associated DNA sequencing
RAPD: Random amplified polymorphic DNA
RFLP: Restriction fragment length polymorphisms
rRNA: Ribosomal RNA
SAM: Sequence alignment/map
SAP: Shrimp Alkaline Phosphatase
SBE: Single-base extension
SNP: Single nucleotide polymorphism
SSR: Simple sequence repeats
