# Supplementary figures and images for: Invasive patterns of Biomphalaria straminea revealed by genetic mapping in the Greater Bay Area, China
Source: Infect Dis Poverty. 2026 Jan 15;15:9. doi: 10.1186/s40249-025-01411-8 (PMC12805737; doi:10.1186/s40249-025-01411-8)

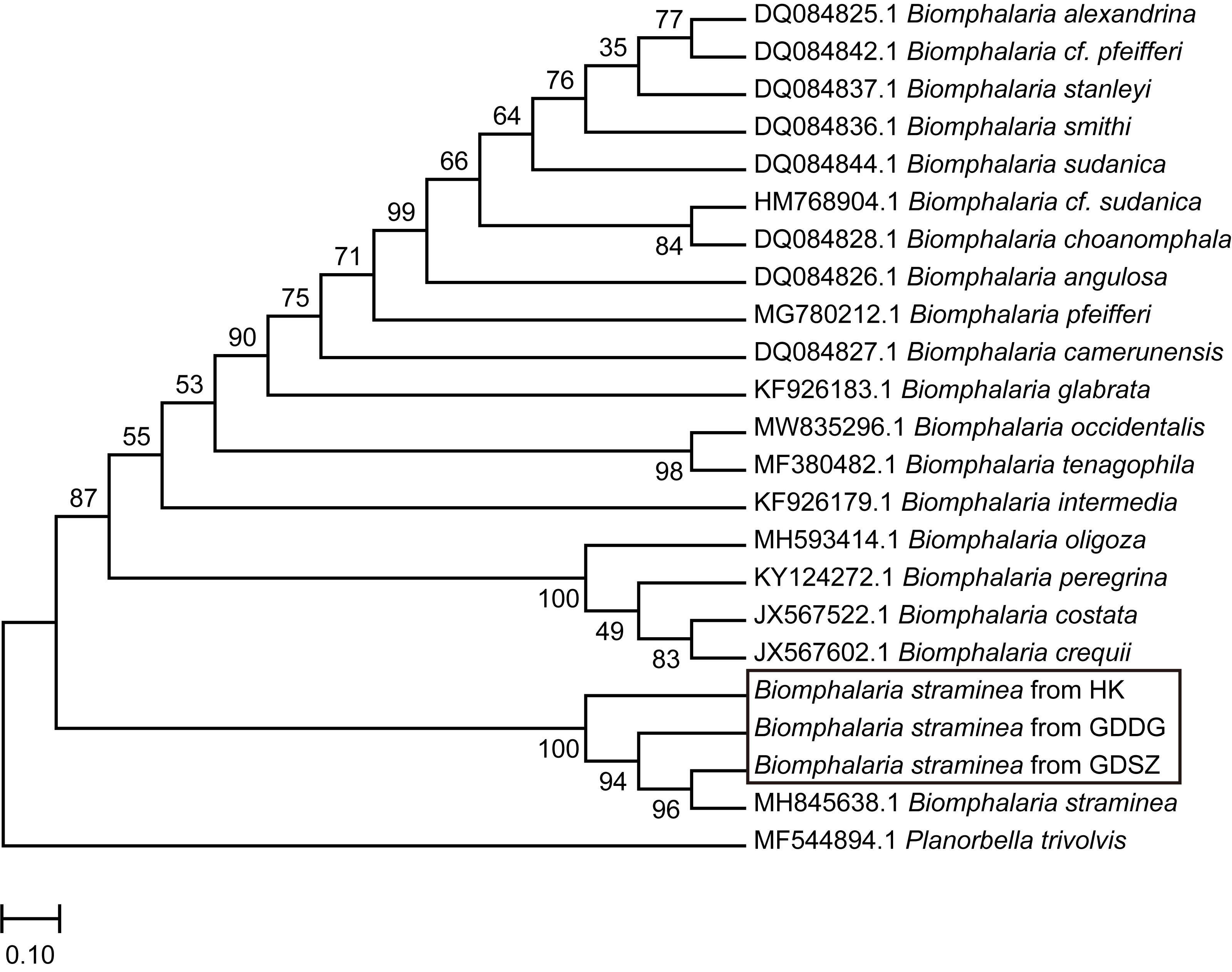

Supplement: Supplementary file 3 — Supplementary material 3. The primers used in the iPLEX assay [file 40249_2025_1411_MOESM3_ESM.tif]

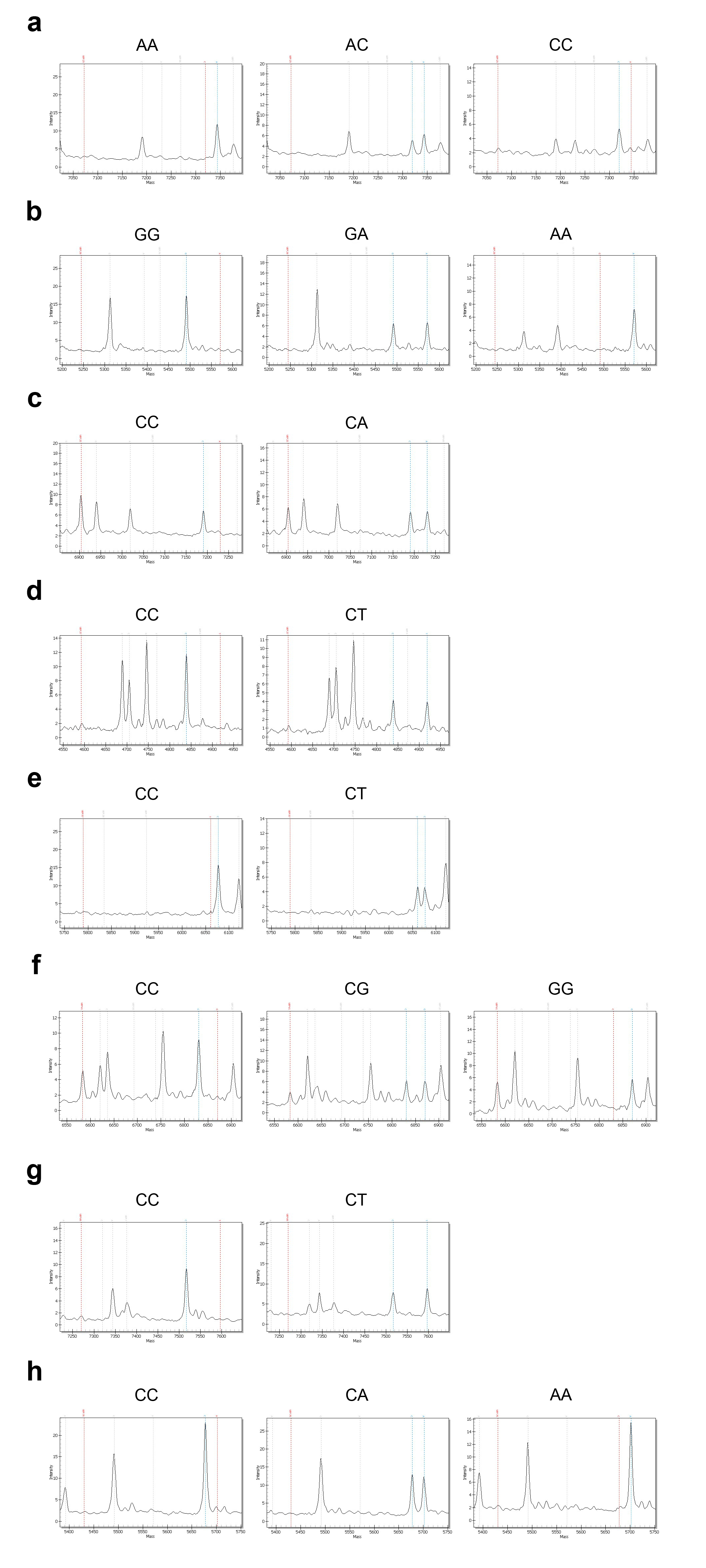

Supplement: Supplementary file 7 — Supplementary material 7. Quality evaluation of reads obtained from ddRAD-seq [file 40249_2025_1411_MOESM7_ESM.tif]

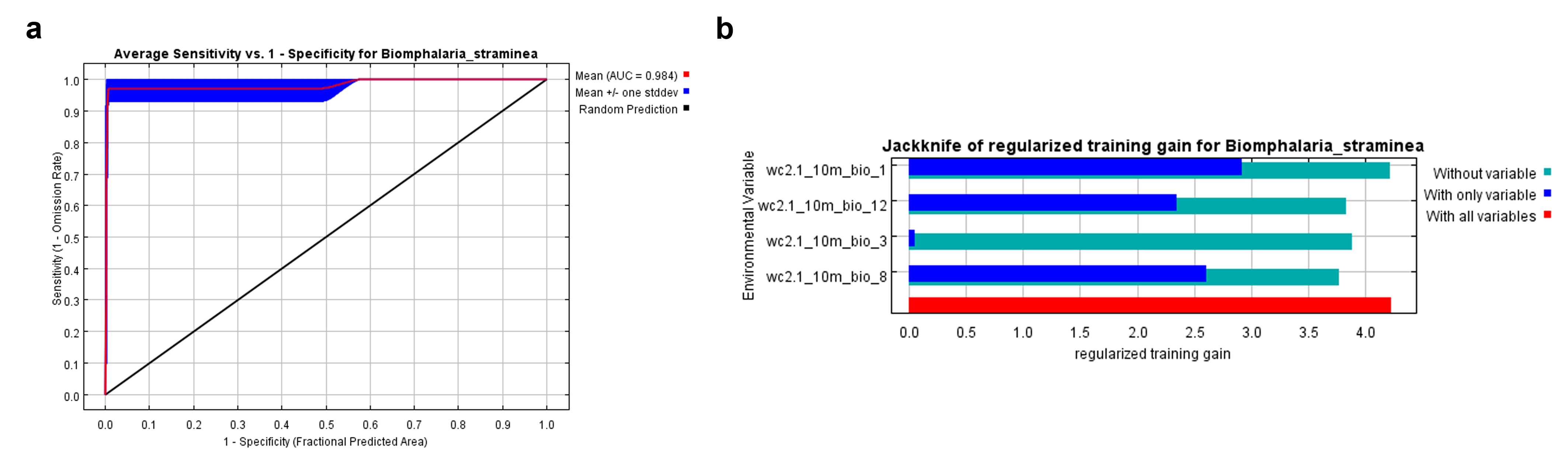

Supplement: Supplementary file 8 — Supplementary material 8. Detailed information of the 80 high-quality SNPs [file 40249_2025_1411_MOESM8_ESM.tif]
